# Finite Difference Methods for Option Pricing under Lévy Processes: Wiener-Hopf Factorization Approach

**DOI:** 10.1155/2013/963625

**Published:** 2013-12-30

**Authors:** Oleg Kudryavtsev

**Affiliations:** ^1^Department of Informatics, Russian Customs Academy Rostov Branch, Budennovskiy 20, Rostov-on-Don 344002, Russia; ^2^Faculty of Mathematics, Mechanics and Computer Science, Southern Federal University, Miltchakova 8A, Rostov-on-Don 344090, Russia

## Abstract

In the paper, we consider the problem of pricing options in wide classes of Lévy processes. We propose a general approach to the numerical methods based on a finite difference approximation for the generalized Black-Scholes equation. The goal of the paper is to incorporate the Wiener-Hopf factorization into finite difference methods for pricing options in Lévy models with jumps. The method is applicable for pricing barrier and American options. The pricing problem is reduced to the sequence of linear algebraic systems with a dense Toeplitz matrix; then the Wiener-Hopf factorization method is applied. We give an important probabilistic interpretation based on the infinitely divisible distributions theory to the Laurent operators in the correspondent factorization identity. Notice that our algorithm has the same complexity as the ones which use the explicit-implicit scheme, with a tridiagonal matrix. However, our method is more accurate. We support the advantage of the new method in terms of accuracy and convergence by using numerical experiments.

## 1. Introduction

In recent years more and more attention has been given to stochastic models of financial markets which depart from the traditional Black-Scholes model. We concentrate on one-factor non-Gaussian exponential Lévy models. These models provide a better fit to empirical asset price distributions that typically have fatter tails than Gaussian ones and can reproduce volatility smile phenomena in option prices. For an introduction to applications of these models applied to finance, we refer to [1, 2].

Option valuation under Lévy processes has been dealt with by a host of researchers; therefore, an exhaustive list is virtually impossible. However, the pricing of barrier options in exponential Lévy models still remains a mathematical and computational challenge (see, e.g., [3–6] for recent surveys of the state of the art of exotic option pricing in Lévy models).

The most general method to price barrier or American options deals with solving the corresponding partial integrodifferential equation (the generalized Black-Scholes equation) with appropriate boundary conditions. Note that in the case of American options free boundary problem arises. There are four main numerical methods for solving the partial integrodifferential equation (PIDE): multinomial trees, finite difference schemes, Galerkin methods, and numerical Wiener-Hopf factorization methods.

In [7], A family of Markov chain approximations of jump-diffusion models is constructed. Multinomial trees can be considered as special cases of explicit finite difference schemes. The main advantage of the method is simplicity of implementation; the drawbacks are inaccurate representation of the jumps and slow convergence.

Galerkin methods are based on the variational formulation of PIDE. While implementation of finite difference methods requires only a moderate programming knowledge, Galerkin methods use specialized toolboxes. Finite difference schemes use less memory than Galerkin methods, since there is no overhead for managing grids, but a refinement of the grid is more difficult. A wavelet Galerkin method for pricing American options under exponential Lévy processes is constructed in [8]. A general drawback of variational methods is that, for processes of finite variation, the convergence can be proved in the *H* ^*s*^-norm only, where *s* < 1/2; hence, the convergence in *C*-norm is not guaranteed.

In a finite difference scheme, derivatives are replaced by finite differences. In the presence of jumps, one needs to discretize the integral term as well. Finite difference schemes were applied to pricing of continuous barrier options in [9] and to pricing of American options in [10–12].

A construction of any finite difference scheme involves discretization in space and time, truncation of large jumps, and approximation of small jumps. Truncation of large jumps is necessary because an infinite sum cannot be calculated; approximation of small jumps is needed when Lévy measure diverges at zero. The result is a linear system that needs to be solved at each time step, starting from payoff function. In the general case, solution of the system on each time step by a linear solver requires *O*(*m* ^2^) operations (*m* is a number of space points), which is too time consuming. In [9–11], the integral part is computed using the solution from the previous time step, while the differential term is treated implicitly. This leads to the explicit-implicit scheme, with tridiagonal system which can be solved in *O*(*m*ln⁡⁡*m*) operations. The paper [12] uses the implicit scheme and the iteration method at each time step. The methods in [10–12] are applicable to processes of infinite activity and finite variation; the part of the infinitesimal generator corresponding to small jumps is approximated by a differential operator of first order (additional drift component). The paper [9] uses an approximation by a differential operator of second order (additional diffusion component).

It follows from the analysis of the above methods for option pricing that in general case finite difference schemes seem to be the best choice. However, the essential disadvantage of the existing methods is speed and/or accuracy.

In [5], the fast and accurate numerical method for pricing barrier option in a wide class of Lévy processes was developed. The fast Wiener-Hopf factorization method (FWHF method) constructed in the paper is based on an efficient approximation of the Wiener-Hopf factors in the exact formula for the solution and the fast Fourier transform algorithm. In contrast to finite difference methods where the application entails an analysis of the underlying Lévy model, the FWHF method deals with the characteristic exponent of the process.

The method in [5] uses the interpretation of the factors as the expected present value operators (EPV operators)—integral operators suggested in [13] which calculate the (discounted) expected present values of streams of payoffs under supremum and infimum processes. This interpretation allows one to guess the optimal exercise boundary quite naturally and give a simple proof of optimality; see details in [14].

The goal of the paper is to incorporate the Wiener-Hopf factorization into finite difference methods for pricing options in Lévy models with jumps in terms of Laurent and Toeplitz matrices. The theory of Laurent and Toeplitz operators allows for solving linear algebraic systems related to the finite difference schemes sufficiently fast and accurate. Moreover, the correspondent matrix operators also admit probabilistic interpretation as expectation operators and they have similar properties to the ones of EPV-operators in [14]. It allows for developing effective methods for solving many standard problems of option pricing.

The method presented in the paper combines speed, simplicity, and accuracy. As our numerical examples show that it is rather faster than existing finite difference schemes. We generalize accurate finite difference scheme developed in [12] on processes of order more than 1 and describe the outline of the solution to the standard problems of option pricing.

The rest of the paper is organized as follows. In Section 2, we give necessary definitions of the theory of Lévy processes. In Section 3 we consider model problems related to the option pricing which can be reduced to solving Toeplitz systems. We provide the formulas for Wiener-Hopf factorization in terms of Laurent matrices and give the probabilistic interpretation to the factors.  Section 4 incorporates the Wiener-Hopf factorization of Toeplitz matrices into finite difference methods for pricing barrier and American options. In Section 5, we produce numerical examples and compare several methods for pricing barrier and American options.  Section 6 concludes the paper.

## 2. Lévy Processes: General Definitions

A Lévy process is a stochastically continuous process with stationary independent increments (for general definitions, see, e.g., [15]). A Lévy process may have a Gaussian component and/or pure jump component. The latter is characterized by the density of jumps, which is called the Lévy density. We denote it by *F*(*dy*). A Lévy process can be completely specified by its characteristic exponent, *ψ*, definable from the equality *E*[*e* ^*iξX*(*t*)^] = *e* ^−*tψ*(*ξ*)^ (we confine ourselves to the one-dimensional case). The characteristic exponent is given by the Lévy-Khintchine formula:

(1) $$\begin{array}{c} \psi \left(\xi \right)=\frac{\sigma^{2}}{2}\xi^{2}-i\mu \xi +\overset{+\infty }{\underset{-\infty }{\int }}\left(1-e^{i\xi y}+{i\xi y1}_{|y|\leq 1}\right)F\left(dy\right), \end{array}$$

where *σ* ^2^ and *μ* are the variance and drift coefficient of the Gaussian component and *F*(*dy*) satisfies

(2) $$\begin{array}{c} \int_{R\setminus \{0\}}\min\left\{1,y^{2}\right\}F\left(dy\right)<+\infty . \end{array}$$

Assume that the riskless rate *r* is constant, and, under a risk-neutral measure chosen by the market, the underlying asset evolves as *S*_*t*_ = *S*_0_ *e* ^*X*_*t*_^, where *X*_*t*_ is a Lévy process. Then we must have *E*[*e* ^*X*_*t*_^]<+*∞*, and, therefore, *ψ* must admit the analytic continuation into the strip *Im*⁡ *ξ* ∈ (−1,0) and continuous continuation into the closed strip *Im*⁡ *ξ* ∈ [−1,0]. Further, the following condition (the EMM requirement) must hold: *E*[*e* ^*X*_*t*_^] = *e* ^*rt*^. Equivalently,

(3) $$\begin{array}{c} r+\psi \left(-i\right)=0, \end{array}$$

where *r* is instantaneous interest rate. The latter condition determines the drift via the other parameters of the Lévy process:

(4) $$\begin{array}{c} \mu =r-\frac{\sigma^{2}}{2}+\overset{+\infty }{\underset{-\infty }{\int }}\left(1-e^{y}+{y1}_{|y|\leq 1}\right)F\left(dy\right). \end{array}$$

Hence, the characteristic exponent may be rewritten as follows:

(5) ψ(ξ)=σ22ξ2−i(r−σ22)ξ +∫−∞+∞(1−eiξy−iξ(1−ey))F(dy).

Then the infinitesimal generator of *X* (denote it by) *L* is an integrodifferential operator which acts as follows:

(6) Lu(x)=σ22u′′(x)+(r−σ22)u′(x) +∫−∞+∞(u(x+y)−u(x)−(ey−1)u′(x))F(dy).

In empirical studies of financial markets, the following classes of Lévy processes are popular: the Merton model [16], double-exponential jump-diffusion model (DEJD) introduced to finance by Lipton [17] and Kou [18], generalization of DEJD model constructed by Levendorskiǐ [19] and labeled later hyperexponential jump-diffusion model (HEJD), variance gamma processes (VGP) introduced to finance by Madan with coauthors (see, e.g., [20]), hyperbolic processes constructed in [21, 22], normal inverse Gaussian processes constructed by Barndorff-Nielsen [23] and generalized in [24], and extended Koponen's family introduced in [25, 26] and labeled KoBoL model in [1]. Koponen [27] introduced a symmetric version; Boyarchenko and Levendorskiǐ [25, 26] gave a nonsymmetric generalization; later, in [28], a subclass of this model appeared under the name CGMY model.

**Example 1**

The characteristic exponent of a pure jump KoBoL process of order *ν* ∈ (0,2), *ν* ≠ 1, is given by

(7) ψ(ξ)=−iμξ+cΓ(−ν)[λ+ν−(λ++iξ)ν       +(−λ−)ν−(−λ−−iξ)ν],

where *c* > 0, *μ* ∈ **R**, and *λ*_−_ < −1 < 0 < *λ*_+_. Formula (7) is derived in [1, 25] from the Lévy-Khintchine formula with the Lévy densities of negative and positive jumps, *F*_∓_(*dy*), given by

(8) $$\begin{array}{c} F_{\mp }\left(dy\right)=ce^{\lambda_{\pm }y}{\left|y\right|}^{-\nu -1}dy. \end{array}$$

**Example 2**

In DEJD model, *F*_∓_(*dy*) are given by exponential functions on negative and positive axis, respectively:

(9) $$\begin{array}{c} F_{\mp }\left(dy\right)=c_{\pm }\left(\pm \lambda_{\pm }\right)e^{\lambda_{\pm }y}, \end{array}$$

where *σ* > 0, *μ* ∈ **R**, *c*_±_ > 0, and *λ*_−_ < −1 < 0 < *λ*_+_. Then the characteristic exponent is of the form

(10) $$\begin{array}{c} \psi \left(\xi \right)=\frac{\sigma^{2}}{2}\xi^{2}-i\mu \xi +\frac{ic_{+}\xi }{\lambda_{+}+i\xi }+\frac{ic_{-}\xi }{\lambda_{-}+i\xi }. \end{array}$$

## 3. Wiener-Hopf Factorization for Finite Difference Schemes 

### 3.1. Wiener-Hopf Factorization for Finite Difference Schemes: Problems with a Barrier

Notice that many option pricing problems with a barrier can be reduced to the family of the following problems:

(11) $$\begin{array}{c} q^{-1}\left(q-L\right)g\left(x\right)=G\left(x\right),\;x>0,\\ g\left(x\right)=0,\;x\leq 0, \end{array}$$

where *q* > 0.

Choose a space step Δ*x*, and set *x*_*l*_ = *l*Δ*x*, *l* ∈ **Z**. Fix *q* > 0 and apply any finite difference scheme to (11) (see, e.g., [9, 11, 12]), which may be reduced to approximate the infinitesimal generator *L* as follows (the example of the reduction can be found in [12]):

(12) $$\begin{array}{c} Lg\left(x_{k}\right)=\underset{l\neq 0}{\sum }\alpha_{l}g\left(x_{k+l}\right)-\underset{l\neq 0}{\sum }\alpha_{l}g\left(x_{k}\right), \end{array}$$

where

(13) $$\begin{array}{c} \underset{l\neq 0}{\sum }\alpha_{l}<\infty ,\;\alpha_{l}>0,\;\;l\neq 0. \end{array}$$

Then we can approximate *q* ^−1^(*q* − *L*) as follows:

(14) $$\begin{array}{c} q^{-1}\left(q-L\right)g\left(x_{k}\right)=\underset{l\in Z}{\sum }a_{l}g\left(x_{k-l}\right), \end{array}$$

(15) $$\begin{array}{c} a_{l}=-q^{-1}\alpha_{-l},\;l\neq 0,\;a_{0}=1+q^{-1}\underset{l\neq 0}{\sum }\alpha_{l}. \end{array}$$

The sequence {*a*_*l*_}_*l*=−*∞*_^+*∞*^ generates doubly infinite Laurent matrix *L*(*a*) which is constant along the diagonals:

(16) $$\begin{array}{c} L\left(a\right)=\left(\begin{array}{cccccc} \cdots & \cdots & \cdots & \cdots & \cdots & \cdots \\ \cdots & a_{0} & a_{-1} & a_{-2} & a_{-3} & \cdots \\ \cdots & a_{1} & a_{0} & a_{-1} & a_{-2} & \cdots \\ \cdots & a_{2} & a_{1} & a_{0} & a_{-1} & \cdots \\ \cdots & a_{3} & a_{2} & a_{1} & a_{0} & \cdots \\ \cdots & \cdots & \cdots & \cdots & \cdots & \cdots \end{array}\right). \end{array}$$

After the discretization, the function *g* ∈ *L*_2_(**R**) turns into a piecewise constant function. Thus, we may consider *g* = (…, *g*(*x*_−2_), *g*(*x*_−1_), *g*(*x*_0_), *g*(*x*_1_), *g*(*x*_2_),…) as an element of *l*_2_(**Z**). Then we may rewrite (14) as follows:

(17) $$\begin{array}{c} q^{-1}\left(q-L\right)g\left(x_{k}\right)={\left(L\left(a\right)g\right)}_{k},\;k\in Z. \end{array}$$

Let **T** stand for the complex unit circle. Since {*a*_*l*_} belongs to *l*_1_(**Z**), we may introduce the function *a*(*t*) = ∑_*k*_ *a*_*k*_ *t* ^*k*^, *t* ∈ **T**, which is known as the symbol of the Laurent matrix or of the Laurent operator *L*(*a*). Recall that the family of all functions with absolutely converging Fourier series is the Wiener algebra *W* : = *W*(**T**) (see details in [29]), which is a Banach algebra with respect to pointwise multiplication of functions and the norm ||*a*||_*W*_ = ∑_*k*_ | *a*_*k*_|.

Further, the sequence {*a*_*k*_}_*k*=−*∞*_^+*∞*^ is the sequence of the Fourier coefficients of *a*(*t*):

(18) $$\begin{array}{c} a_{k}=\frac{1}{2\pi }\overset{2\pi }{\underset{0}{\int }}a\left(e^{i\varphi }\right)e^{-ik\varphi }d\varphi ,\;k\in Z. \end{array}$$

Denote by *F* : *L*_2_(**T**) → *l*_2_(**Z**) the operator which maps a function *a*(*t*) to the sequence of its Fourier coefficients {*a*_*k*_}_*k*=−*∞*_^+*∞*^ (see (18)).

It is well known that the Laurent matrix *L*(*a*) is the matrix representation of the multiplication by *a*(*t*) operator on *L*_2_(**T**) with respect to the orthonormal basis $\left\{(1/\sqrt{2\pi })e^{ik\varphi }\right\}$. Hence, we have

(19) $$\begin{array}{c} L\left(a\right)=FaF^{-1}. \end{array}$$

It follows from (19) that

(20) $$\begin{array}{c} L\left(a_{1}\right)L\left(a_{2}\right)=L\left(a_{1}a_{2}\right),\;\forall a_{1},a_{2}\in L_{\infty }\left(T\right). \end{array}$$

According to the Wiener theorem, (see, e.g., [29]) if *a* ∈ *W* and *a*(*t*) ≠ 0, ∀*t* ∈ **T**, then *a* ^−1^ = 1/*a* ∈ *W*. Let us denote by *GW* the set of all invertible elements of the algebra *W*. It follows from (20) that if *a* ∈ *GW* then the matrix *L*(*a*) is invertible with the inverse *L*(*a* ^−1^).

The sequence {*a*_*l*_}_*l*=−*∞*_^+*∞*^ also generates the infinite Toeplitz matrix *T*(*a*):

(21) $$\begin{array}{c} T\left(a\right)=\left(\begin{array}{ccccc} a_{0} & a_{-1} & a_{-2} & a_{-3} & \cdots \\ a_{1} & a_{0} & a_{-1} & a_{-2} & \cdots \\ a_{2} & a_{1} & a_{0} & a_{-1} & \cdots \\ a_{3} & a_{2} & a_{1} & a_{0} & \cdots \\ \cdots & \cdots & \cdots & \cdots & \cdots \end{array}\right). \end{array}$$

When a finite difference approximation is applied (see (14)), (11) may be rewritten as follows:

(22) $$\begin{array}{c} {\left(L\left(a\right)g\right)}_{k}=G_{k},\;k\in N,\\ g_{k}=0,\;k\in Z,\;\;k\leq 0, \end{array}$$

where *G*_*k*_ = *G*(*x*_*k*_). Let *P* denote the orthogonal projection of *l*_2_(**Z**) onto *l*_2_(**N**):

(23) $$\begin{array}{c} Pu_{k}=\{\begin{array}{cc} u_{k}, & k>0,\\ 0, & k\leq 0. \end{array} \end{array}$$

Then, taking into account that *T*(*a*) = *PL*(*a*)*P*, we rewrite (22) in terms of Toeplitz matrices:

(24) $$\begin{array}{c} T\left(a\right)g=PG, \end{array}$$

where *G* = (…, *G*(*x*_−2_), *G*(*x*_−1_), *G*(*x*_0_), *G*(*x*_1_), *G*(*x*_2_),…) is considered as an element of *l*_2_(**Z**).

The standard theory of Toeplitz matrices (see details in Section 1.5, [29]) leads us to the following theorem.

**Theorem 3**

Let a function *a* ∈ *W* be able to be represented in the form

(25) $$\begin{array}{c} a=\exp\left(b\right),\;b\in W. \end{array}$$

Then the operator *T*(*a*) is invertible and there exist *a*_+_, *a*_−_ ∈ *GW* such that

(26) $$\begin{array}{c} a_{+}\left(t\right)=\underset{k\geq 0}{\sum }a_{k}^{+}t^{k},\;t\in T, \end{array}$$

(27) $$\begin{array}{c} a_{-}\left(t\right)=\underset{k\leq 0}{\sum }a_{k}^{-}t^{k},\;t\in T, \end{array}$$

(28) $$\begin{array}{c} a=a_{+}a_{-}, \end{array}$$

(29) $$\begin{array}{c} T\left(a\right)=T\left(a_{-}\right)T\left(a_{+}\right), \end{array}$$

(30) $$\begin{array}{c} T{\left(a\right)}^{-1}=T\left(a_{+}^{-1}\right)T\left(a_{-}^{-1}\right). \end{array}$$

Notice that the identities (29) and (28) are called a Wiener-Hopf factorization for Toeplitz matrices and Wiener functions, respectively.

In the context of the finite difference schemes under consideration one can prove the following proposition.

**Proposition 4**

Let {*a*_*l*_}_*l*=−*∞*_^+*∞*^ defined by (13) and (15) be the sequence of the Fourier coefficients of a *a*(*t*). Then *a*(*t*) satisfies conditions of Theorem 3.

**Proof**

Set

(31) $$\begin{array}{c} \tilde{a}\left(t\right)=1-\frac{a\left(t\right)}{a_{0}},\;t\in T, \end{array}$$

then we have

(32) $$\begin{array}{c} \tilde{a}\left(t\right)=\underset{l\neq 0}{\sum }{\tilde{a}}_{l}t^{l},\;{\tilde{a}}_{l}=-\frac{a_{l}}{a_{0}}. \end{array}$$

According to (15) and (32), there exists a positive number *r*_0_ < 1 such that

(33) $$\begin{array}{c} {||\tilde{a}\left(t\right)||}_{W}<r_{0},\;\forall t\in T. \end{array}$$

Hence, the Taylor series for $\ln(1-\tilde{a}(t))$ (ln⁡⁡(·) is the principal branch of the logarithm),

(34) $$\begin{array}{c} -\underset{n>0}{\sum }\frac{\tilde{a}{\left(t\right)}^{n}}{n} \end{array}$$

converges at every point *t* ∈ **T**.

Set

(35) $$\begin{array}{c} b\left(t\right)=\ln\left(1-\tilde{a}\left(t\right)\right)+\ln\left(a_{0}\right), \end{array}$$

(36) $$\begin{array}{c} \phi_{k}\left(t\right)=-\overset{k}{\underset{n=1}{\sum }}\frac{\tilde{a}{\left(t\right)}^{n}}{n}+\ln\left(a_{0}\right),\;k\in N. \end{array}$$

Because $\tilde{a}(t)\in W$ and the condition (33) is satisfied, we conclude that *ϕ*_*k*_(*t*) is a fundamental sequence which is contained in the Wiener algebra *W*. In fact, for any *ϵ* > 0, there exists a natural number *k* such that

(37) $$\begin{array}{c} {||\phi_{k+m}-\phi_{k}||}_{W}\leq \overset{k+m}{\underset{n=k+1}{\sum }}\frac{r_{0}^{n}}{n}\leq \frac{r_{0}^{k+1}}{\left(k+1\right)\left(1-r_{0}\right)}<\epsilon ,\;\forall m\in N. \end{array}$$

Thus, we have proved that the function *b*(*t*) belongs to the Wiener algebra *W*. Obviously, *a*(*t*) = exp⁡(*b*(*t*)), ∀*t* ∈ **T**.

Let a function *a*(*t*) satisfy the conditions of Proposition 4. Set

(38) $$\begin{array}{c} p\left(t\right)={\left(a\left(t\right)\right)}^{-1},\\ p^{+}\left(t\right)={\left(a_{+}\left(t\right)\right)}^{-1},\\ p^{-}\left(t\right)={\left(a_{-}\left(t\right)\right)}^{-1}. \end{array}$$

From Theorem 3 and Proposition 4, we deduce

(39) $$\begin{array}{c} p\left(t\right)=\underset{l\in Z}{\sum }p_{l}t^{l},\;p\in W, \end{array}$$

(40) $$\begin{array}{c} p^{-}\left(t\right)=\overset{l=0}{\underset{l=-\infty }{\sum }}p_{l}^{-}t^{l},\;p^{-}\in W, \end{array}$$

(41) $$\begin{array}{c} p^{+}\left(t\right)=\overset{l=+\infty }{\underset{l=0}{\sum }}p_{l}^{+}t^{l},\;p^{+}\in W. \end{array}$$

Further, we will describe an algorithm for finding coefficients {*p*_*l*_},  {*p*_*l*_^±^}, based on the theory from [29]. By Theorem 3,

(42) $$\begin{array}{c} L\left(p\right)=L{\left(a\right)}^{-1},\\ T\left(p^{+}\right)=T{\left(a_{+}\right)}^{-1},\\ T\left(p^{-}\right)=T{\left(a_{-}\right)}^{-1}. \end{array}$$

It follows that {*p*_*l*_} is the sequence of the Fourier coefficients of *a* ^−1^. We have

(43) $$\begin{array}{c} p_{k}=\frac{1}{2\pi }\overset{\pi }{\underset{-\pi }{\int }}a{\left(e^{i\varphi }\right)}^{-1}e^{-ik\varphi }d\varphi ,\;k\in Z, \end{array}$$

due to (18).

Wiener-Hopf factorization formula (28) gives

(44) $$\begin{array}{c} p\left(t\right)=p^{+}\left(t\right)p^{-}\left(t\right),\;\forall t\in T. \end{array}$$

The factors *p* ^±^ can be found as follows. From Proposition 4, there exists a function

(45) $$\begin{array}{c} b\left(t\right)=\overset{+\infty }{\underset{k=-\infty }{\sum }}b_{k}t^{k},\;\overset{+\infty }{\underset{k=-\infty }{\sum }}\left|b_{k}\right|<\infty , \end{array}$$

such that

(46) $$\begin{array}{c} b\left(t\right)=\ln a\left(t\right),\;t\in T, \end{array}$$

where the sequence of the Fourier coefficients of this function is defined as

(47) $$\begin{array}{c} b_{k}=\frac{1}{2\pi }\overset{\pi }{\underset{-\pi }{\int }}\ln a\left(e^{i\varphi }\right)e^{-ik\varphi }d\varphi ,\;k\in Z. \end{array}$$

Notice that *p*(*t*) = *e* ^−*b*(*t*)^,  *t* ∈ **T**. Next we define *b*_±_ as

(48) b−(t)=∑k=−∞k=−1bk(tk−1),b+(t)=∑k=1k=+∞bk(tk−1),   t∈T,

Further, we set

(49) $$\begin{array}{c} p_{+}\left(t\right)=e^{-b_{+}(t)},\;p_{-}\left(t\right)=e^{-b_{-}(t)},\;t\in T. \end{array}$$

Finally, we have

(50) $$\begin{array}{c} p_{k}^{\pm }=\frac{1}{2\pi }\overset{\pi }{\underset{-\pi }{\int }}p_{\pm }\left(e^{i\varphi }\right)e^{-ik\varphi }d\varphi ,\;k\in Z. \end{array}$$

Obviously, *p*_*k*_^+^ = 0 as *k* < 0, and *p*_*k*_^−^ = 0 as *k* > 0.

**Remark 5**

Notice that the Wiener-Hopf factorization (44) is also satisfied if we substitute *b*_−_(*t*) + *C* and *b*_+_(*t*) − *C* into (49) (with any constant *C*) instead *b*_−_(*t*) and *b*_+_(*t*), respectively.

Hence, to solve the problem (24), one needs to construct the inverse Toeplitz operator *T*(*a*)^−1^ by using the above algorithm. Thus, an approximate solution to the problem (11) can be written as

(51) $$\begin{array}{c} g=T\left(a_{+}^{-1}\right)T\left(a_{-}^{-1}\right)G. \end{array}$$

From a practical point of view, it is more convenient to rewrite (51) in terms of Laurent operators *L*(*p* ^±^):

(52) $$\begin{array}{c} g=L\left(p_{+}\right)PL\left(p_{-}\right)G. \end{array}$$

An efficient numerical realization of (52) is available by means of fast Fourier transform (FFT) due to (19). The complexity of the method is *O*(*M*ln⁡*M*), where *M* is the number of space discretization points.

### 3.2. Wiener-Hopf Method for Finite Difference Schemes: Optimization Problems

Optimal stopping problems play a very important role in the mathematical finance and they are connected with pricing American, Bermudan, and other types of options. Pricing such options can be typically reduced to the sequence of the following problems.

Let *G*(*x*) be a monotonically increasing function, and it changes sign on the real line. Consider the following problem:  

(53) $$\begin{array}{c} q^{-1}\left(q-L\right)g\left(x\right)=G\left(x\right),\;x>h,\\ g\left(x\right)=0,\;\;x\leq h, \end{array}$$

where the continuous function *g*(*x*) is maximized over barriers *h*.  

Applying a finite difference scheme to (53) which approximates *q* ^−1^(*q* − *L*) by formulas (14) and (15), we obtain the following discrete equation on the half line:

(54) $$\begin{array}{c} {\left(L\left(a\right)g\right)}_{k}=G^{k},\;k>k_{0},\\ g_{k}=0,\;k\leq k_{0}, \end{array}$$

where *g*_*k*_ = *g*(*x*_*k*_), *G* ^*k*^ = (*q*Δ*t*)^−1^ *G*(*x*_*k*_), *k*_0_ maximizes *g*, and *a* is the symbol of a Laurent *L*(*a*) (see (12)–(16)).

Introduce the orthogonal projection *P*_*l*_ as follows:

(55) $$\begin{array}{c} P_{l}u_{k}=\{\begin{array}{cc} u_{k}, & k>l,\\ 0, & k\leq l. \end{array} \end{array}$$

Further, we factorize the corresponding Toeplitz operator *T*(*a*) (see Theorem 3, Proposition 4, and (38)–(50)). The factorization formulas (48) are chosen in such a way that functions *p*, *p* ^+^, and *p* ^−^ are characteristic functions of infinitely divisible distributions.

**Theorem 6**

Let sequences {*p*_*l*_}, {*p*_*l*_^±^} be as defined by (43) and (47)–(50), and assume that the conditions of Proposition 4 are satisfied. Set

(56) $$\begin{array}{c} P\left(\xi \right)=\underset{l\in Z}{\sum }p_{l}\exp\left(-il\xi \Delta x\right),\;\xi \in R,\\ P^{+}\left(\xi \right)=\overset{l=0}{\underset{l=-\infty }{\sum }}p_{l}^{-}\exp\left(-il\xi \Delta x\right),\;\xi \in R,\\ P^{-}\left(\xi \right)=\overset{l=+\infty }{\underset{l=0}{\sum }}p_{l}^{+}\exp\left(-il\xi \Delta x\right),\;\xi \in R. \end{array}$$

Then *P*, *P* ^+^, and *P* ^−^ are characteristic functions of infinitely divisible lattice distributions supported on {*x* = *k*Δ*x* | *k* ∈ **Z**}, {*x* = *k*Δ*x* | *k* ∈ **Z**, *k* ≥ 0}, and {*x* = *k*Δ*x* | *k* ∈ **Z**, *k* ≤ 0}, respectively.

**Proof**

Recall that the characteristic function of an infinitely divisible lattice distribution with the maximal step Δ*x* has the following form (see, e.g., [30]):

(57) $$\begin{array}{c} c\left(\xi \right)=\exp\left(\underset{k\in Z}{\sum }c_{k}\left(\exp\left(ik\xi \Delta x\right)-1\right)\right),\;\;\xi \in R, \end{array}$$

where *c*_*k*_ ≥ 0, ∀*k* ∈ **Z**,  ∑_*k*∈**Z**_ *c*_*k*_ < +*∞*.

From (35), we have

(58) $$\begin{array}{c} -b\left(t\right)=\overset{+\infty }{\underset{n=1}{\sum }}\frac{\tilde{a}{\left(t\right)}^{n}}{n}-\ln\left(a_{0}\right). \end{array}$$

Since *b* ∈ *W* and *b*(1) = 0, the function −*b* can be written as follows:

(59) $$\begin{array}{c} -b\left(t\right)=\overset{+\infty }{\underset{k=-\infty }{\sum }}\left(-b_{k}\right)\left(t^{k}-1\right), \end{array}$$

where *b*_*k*_ are defined by (47).

Notice that by the definition (see (32)) ${\tilde{a}}_{k}>0$, *k* ≠ 0. It follows that the Fourier coefficients of $\tilde{a}{\left(t\right)}^{n}/n$ are also positive for every *n* due to Cauchy's series product theorem. Since ${||\tilde{a}||}_{W}<r_{0}<1$, ${||{\tilde{a}}^{n}/n||}_{W}<r_{0}^{n}/n$. Hence, all the coefficients −*b*_*k*_ in the formula (59) are positive.

Clearly, the functions *f*(*t*) ∈ *W* (*t* = *e* ^*iϕ*^ ∈ **T**) are continuous on **T** and, when regarded as functions *f*(*e* ^−*i*Δ*xξ*^), *ξ* ∈ **R**, they are (2*π*/Δ*x*)-periodic continuous functions. Hence, the function *p*(*t*) (see (39) and (43)) can be rewritten in the form (57):

(60) $$\begin{array}{c} P\left(\xi \right)=\exp\left(\underset{l\neq 0}{\sum }\left(-b_{-l}\right)\left(\exp\left(il\xi \Delta x\right)-1\right)\right). \end{array}$$

Analogously, we rewrite *P*_±_(*ξ*) in the form (57):

(61) $$\begin{array}{c} P_{-}\left(\xi \right)=\exp\left(\underset{l<0}{\sum }\left(-b_{-l}\right)\left(\exp\left(il\xi \Delta x\right)-1\right)\right),\\ P_{+}\left(\xi \right)=\exp\left(\underset{l>0}{\sum }\left(-b_{-l}\right)\left(\exp\left(il\xi \Delta x\right)-1\right)\right). \end{array}$$

It follows that *P*, *P* ^+^, and *P* ^−^ are characteristic functions of infinitely divisible lattice distributions with the maximal step Δ*x* supported on {*x* = *k*Δ*x* | *k* ∈ **Z**}, {*x* = *k*Δ*x* | *k* ∈ **Z**, *k* ≥ 0}, and {*x* = *k*Δ*x* | *k* ∈ **Z**, *k* ≤ 0}, respectively.

From Theorem 6 we have that there exist discrete random variables *X*, *X* ^+^, and *X* ^−^ taking on values of the form *x*_*k*_ = *k*Δ*x*, *k* ∈ **Z**, such that

(62) $$\begin{array}{c} P\left(X=x_{k}\right)=p_{-k},\;k\in Z,\\ P\left(X^{-}=x_{k}\right)=p_{-k}^{+},\;k\in Z,\\ P\left(X^{+}=x_{k}\right)=p_{-k}^{-},\;k\in Z, \end{array}$$

where {*p*_*k*_} and {*p*_*k*_^±^} are defined by (43) and (47)–(50).

It follows that the corresponding Laurent operators *L*(*p*), *L*(*p* ^+^), and *L*(*p* ^−^) can be interpreted as expectation operators conditioned on current values of *X*, *X* ^−^, and *X* ^+^, respectively:  

(63) $$\begin{array}{c} L\left(p\right)g\left(x_{k}\right)=\underset{l\in Z}{\sum }p_{l}g\left(x_{k-l}\right)=E\left[g\left(x_{k}+X\right)\right],\\ L\left(p^{+}\right)g\left(x_{k}\right)=\overset{l=+\infty }{\underset{l=0}{\sum }}p_{l}^{+}g\left(x_{k-l}\right)=E\left[g\left(x_{k}+X^{-}\right)\right],\\ L\left(p^{-}\right)g\left(x_{k}\right)=\overset{l=0}{\underset{l=-\infty }{\sum }}p_{l}^{-}g\left(x_{k-l}\right)=E\left[g\left(x_{k}+X^{+}\right)\right]. \end{array}$$

The following simple properties are immediate from the interpretation of *L*(*p*_±_) as expectation operators.

**Proposition 7**

Laurent operators *L*(*p*_±_) enjoy the following properties.

- If *g*_*k*_ = 0,  ∀*k* ≥ *k*_0_, then, ∀*k* ≥ *k*_0_, (*L*(*p*_−_)*g*)_*k*_ = 0.
- If *g*_*k*_ = 0,  ∀*k* ≤ *k*_0_, then, ∀*k* ≤ *k*_0_, (*L*(*p*_+_)*g*)_*k*_ = 0.
- If *g*_*k*_ ≥ 0,  ∀*k*, then, (*L*(*p*_−_)*g*)_*k*_ ≥ 0, ∀*k*. If, in addition, there exists *k*_0_ such that *g*_*k*_ > 0  ∀*k* > *k*_0_, then (*L*(*p*_−_)*g*)_*k*_ > 0,  ∀*k*.
- If *g*_*k*_ ≥ 0,  ∀*k*, then (*L*(*p*_+_)*g*)_*k*_ ≥ 0, ∀*x*. If, in addition, there exists *k*_0_ such that *g*_*k*_ > 0  ∀*k* < *k*_0_, then (*L*(*p*_+_)*g*)_*k*_ > 0,  ∀*k*.
- If *g* = {*g*_*k*_} is monotone, then {(*L*(*p*_−_)*g*)_*k*_} and {(*L*(*p*_+_)*g*)_*k*_} are also monotone.

Proposition 7 is a direct analog of the properties of the expected present value operators introduced in [14]; see Proposition 6.2.1.

Taking into account that *G* = {*G* ^*k*^} in (54) is a monotonically increasing sequence and it changes the sign, then from Proposition 7 an approximate solution *g* = (…, *g*(*x*_−2_), *g*(*x*_−1_), *g*(*x*_0_), *g*(*x*_1_), *g*(*x*_2_),…) to the problem (53) can be written in terms of Laurent operators *L*(*p* ^±^):

(64) $$\begin{array}{c} g=L\left(p_{+}\right)P_{k_{0}}L\left(p_{-}\right)G, \end{array}$$

where the only number *k*_0_ can be found from the following conditions:  

(65) $$\begin{array}{c} {\left(L\left(p_{-}\right)G\right)}_{k}>0,\;k>k_{0},\\ {\left(L\left(p_{-}\right)G\right)}_{k}\leq 0,\;k\leq k_{0}. \end{array}$$

We remark that (64) includes the requirement that the series

(66) $$\begin{array}{c} w_{k}=\overset{l=0}{\underset{l=-\infty }{\sum }}p_{l}^{-}G^{k-l},\;k\in Z \end{array}$$

are convergent. An efficient numerical realization of (64) is based on (19) and fast Fourier transform.

### 3.3. Wiener-Hopf Factorization for Finite Difference Schemes: Algorithm

In the subsection, we give an algorithm of the construction of an approximate Wiener-Hopf factorization for finite difference schemes.

*Wiener-Hopf Factorization*

**Step 1**

Input the interest rate *r* and the parameters of the Lévy exponents (5).

**Step 2**

Input the space step Δ*x*.

**Step 3**

Choose a finite difference scheme (FDS) for an approximation of the infinitesimal generator *L*.

**Step 4**

Choose desired truncation error *ϵ* for coefficients {*α*_*l*_} in (12) (as a rule, the choice *ϵ* = 10^−6^ is optimal). Due to the FDS, calculate *α*_*l*_, *l* = −1, −2,…, *l* ^−^, where *l* ^−^ = max⁡⁡{*l* < 0 | |*α*_*l*_| < *ϵ*/2}; calculate *α*_*l*_, *l* = 1,2,…, *l* ^+^, where *l* ^+^ = min⁡⁡{*l* > 0 | |*α*_*l*_| < *ϵ*/2}. Set *α*_*l*_ = 0, as *l* < *l* ^−^ or *l* > *l* ^+^.

**Step 5**

Input the terminal date *T* and define the number of time steps *n* (the choice of *n* typically depends on the finite difference scheme). Set space step Δ*t* = *T*/*n* and *q* = (Δ*t*)^−1^ + *r*.

**Step 6**

Input *x*_min⁡_ and *x*_max⁡_, the lower and upper bounds for the space variable *x*. As a rule, the choice *x*_min⁡_ = ln⁡⁡(0.4) and *x*_max⁡_ = ln⁡⁡(2.5) is optimal.

**Step 7**

Define the number of space points *m* as follows. Set *l*_0_ = max⁡⁡{−*l*_−_; *l*_+_; (*x*_max⁡_ − *x*_min⁡_)/2Δ*x*}. We find integer number *k*_0_ such that 2^*k*_0_−1^ < *l*_0_ ≤ 2^*k*_0_^ and set *m* = 2^*k*_0_^. We will use fast Fourier transform for real-valued functions (FFT); see details in [5, 31]. That is why we choose the number of space points as a power of 2.

**Step 8**

Find coefficients *a*_*l*_,  *l* = −*m* + 1,…, *m*, by the formula (15).

**Step 9**

Denote by *τ*_*k*_ = exp⁡⁡(*iπk*/*m*),  *k* = −*m* + 1,…, *m*. Find *a*(*τ*_*k*_) = ∑_*l*=−*m*+1_^*l*=*m*^ *a*_*l*_ *τ*_*k*_^*l*^,  *k* = −*m* + 1,…, *m*, using FFT.

**Step 10**

We find the symbol of *L*(*p*) = (*L*(*a* ^−1^)): *p*(*τ*_*k*_) = *a* ^−1^(*τ*_*k*_),  *k* = −*m* + 1,…, *m*  (see (39)).

**Step 11**

We find *b*(*τ*_*k*_): = ln⁡⁡(*a*(*τ*_*k*_)), *k* = −*m* + 1,…, *m* (see (46)). Using inverse FFT, we obtain the sequence of coefficients *b*_*k*_,  *k* = −*m* + 1,…, *m*, for decomposition of *b*(*τ*) to the series

(67) $$\begin{array}{c} b\left(\tau \right)=\overset{l=m}{\underset{l=-m+1}{\sum }}b_{l}\tau^{l} \end{array}$$

**Step 12**

Set *b*_0_^−^ = −∑_*l*=−*m*+1_^*l*=−1^ *b*_*l*_ and *b*_−_(*τ*) = ∑_*l*=−*m*+1_^*l*=−1^ *b*_*l*_ *τ* ^*l*^ + *b*_0_^−^; set *b*_0_^+^ = *b*_0_ − *b*_0_^−^ and *b*_+_(*τ*) = ∑_*l*=1_^*l*=*m*^ *b*_*l*_ *τ* ^*l*^ + *b*_0_^+^ (see (48)). Using FFT we obtain *b*_±_(*τ*_*k*_),  *k* = −*m* + 1,…, *m*.

**Step 13**

We find the symbols of *L*(*p* ^±^): *p* ^±^(*τ*_*k*_) = exp⁡(−*b*_±_(*τ*_*k*_)),  *k* = −*m* + 1,…, *m* (see (49)).

## 4. Implementation of Wiener-Hopf Method for Solution to Standard Problems on Option Pricing

We assume that the riskless rate *r* > 0 is constant and, under a risk-neutral measure chosen by the market, the log price of the stock *X*_*t*_ = log⁡*S*_*t*_ follows a Lévy process with the infinitesimal generator *L* (see (6)) and characteristic exponent *ψ* (see (5)).

### 4.1. Barrier Options

Consider a contract which pays the specified amount *G*(*S*_*T*_) at the terminal date *T*, provided during the life-time of the contract, the price of the stock does not cross a specified constant barrier *H* from above (*down-and-out barrier options*) or from below (*up-and-out barrier options*). When the barrier is crossed, the option expires worthless or the option owner is entitled to some* rebate*. We restrict ourselves to the case of down-and-out barrier options without rebate; the generalization to the cases of a up-and-out barrier options and barrier options with rebate is straightforward. The price *V*(*t*, *S*_*t*_) of such barrier option can be found as the solution to the following integrodifferential equation with initial and boundary conditions (see [1]). Set *x* = ln⁡⁡(*S*/*H*), *g*(*x*) = *G*(*He* ^*x*^), and *v*(*t*, *x*) = *V*(*t*, *He* ^*x*^). Then,

(68) $$\begin{array}{c} \left(\partial_{t}+L-r\right)v\left(t,x\right)=0,\;0\leq t\leq T,\;\;x>0, \end{array}$$

(69) $$\begin{array}{c} v\left(T,x\right)=g\left(x\right),\;x>0, \end{array}$$

(70) $$\begin{array}{c} v\left(t,x\right)=0,\;0\leq t\leq T,\;\;x\leq 0. \end{array}$$

The most numerical methods start with a time discretization (the method of lines); see, for example, [5]. Divide [0, *T*] into *n* subperiods by points *t*_*j*_ = *j*Δ*t*, *j* = 0,1,…, *n*, where Δ*t* = *T*/*n*, and denote by *v*_*j*_(*x*) the approximation to *v*(*x*, *t*_*j*_). Then *v*_*n*_(*x*) = *g*(*x*), and by discretizing the derivative ∂_*t*_ in (68), we obtain, for *j* = *n* − 1, *n* − 2,…, 0,

(71) $$\begin{array}{c} \frac{v_{j+1}\left(x\right)-v_{j}\left(x\right)}{\Delta t}-\left(r-L\right)v_{j}\left(x\right)=0,\;x>0. \end{array}$$

Equation (70) assumes the form

(72) $$\begin{array}{c} v_{j}\left(x\right)=0,\;x\leq 0. \end{array}$$

Set *q* = Δ*t* ^−1^ + *r*; then (71) can be rewritten as follows:

(73) $$\begin{array}{c} q^{-1}\left(q-L\right)v_{j}\left(x\right)={\left(q\Delta t\right)}^{-1}v_{j+1}\left(x\right),\;x>0. \end{array}$$

Notice that the sequence of problems (72) and (73) has the form (11). Applying a finite difference scheme to (72) and (73) which approximates *q* ^−1^(*q* − *L*) by formulas (14) and (15), we obtain the discrete problem of the form (22) which can be easily solved by using (52). See details in Section 3.1. One can speed up the calculations by using real-valued FFT and similar tricks as in [5].

### 4.2. American Options

We consider the American put on a stock which pays no dividends; the generalization to the case of a dividend-paying stock and the American call is straightforward. (Moreover, as it is well-known, changing the direction on the line, the unknown function, the riskless rate, and the process, one can reduce the pricing problem for the American call to the pricing problem for the American put).

Let *V*(*t*, *S*_*t*_) be the price of American put with the strike price *K* and the terminal date *T*. Set *x* = ln⁡⁡(*S*/*K*), *g*(*x*) = *K*(1 − *e* ^*x*^), and *v*(*t*, *x*) = *V*(*t*, *Ke* ^*x*^). Assume that the optimal stopping time is of the form *τ*_*B*_′∧*T*, where *τ*_*B*_′ is the hitting time of a closed set *B* ⊂ **R** × (−*∞*, *T*] by the two-dimensional process ${\hat{X}}_{t}=(X_{t},t)$. Set *𝒞* = **R** × [0, *T*)∖*B* (this is the continuation region, where the option remains alive), and consider the following boundary value problem:

(74) $$\begin{array}{c} \left(\partial_{t}+L-r\right)v\left(t,x\right)=0,\;\left(t,x\right)\in 𝒞, \end{array}$$

(75) $$\begin{array}{c} v\left(t,x\right)=g\left(x\right),\;\left(t,x\right)\in B\;\text{or}\;\;t=T, \end{array}$$

(76) $$\begin{array}{c} v\left(t,x\right)\geq {\left(x\right)}^{+},\;t\leq T,\;\;x\in R, \end{array}$$

(77) $$\begin{array}{c} \left(\partial_{t}+L-r\right)v\left(t,x\right)\leq 0,\;t<T,\;\;\left(t,x\right)\notin \bar{𝒞}, \end{array}$$

where *g*(*x*)^+^ : = max⁡{*g*(*x*), 0}.

Under certain regularity conditions (see Theorem 6.1 in [1]), the continuous bounded solution to the free boundary problem (74)–(77) gives the optimal early exercise region, *B*, and the rational option price, *v*.

We apply the Lévy analog of Carr's randomization procedure developed in Section 6.2.2 of [1] for the American put. Normalize the strike price to 1; divide [0, *T*] into *n* subperiods by points *t*_*j*_ = *j*Δ*t*, *j* = 0,1,…, *n*, where Δ*t* = *T*/*n*; and denote by *v*_*j*_(*x*) the approximation to *v*(*x*, *t*_*j*_); *h*_*j*_ denotes the approximation to the early exercise boundary at time *t*_*j*_. Then *v*_*n*_(*x*) = *K*(1 − *e* ^*x*^)^+^, and by discretizing the derivative ∂_*t*_ in (74), we obtain, for *j* = *n* − 1, *n* − 2,…, 0,

(78) $$\begin{array}{c} \frac{v_{j+1}\left(x\right)-v_{j}\left(x\right)}{\Delta t}-\left(r-L\right)v_{j}\left(x\right)=0,\;\;x>h_{j}. \end{array}$$

Equation (75) assumes the form

(79) $$\begin{array}{c} v_{j}\left(x\right)=g\left(x\right),\;x\leq h_{j}. \end{array}$$

The approximation *h*_*j*_ to the early exercise boundary is found so that the *v*_*j*_ is maximal.

Introduce ${\tilde{v}}_{j}(x)=v_{j}(x)-g(x)$ and substitute $v_{j}(x)={\tilde{v}}_{j}(x)+g(x)$ into (78) and (79):

(80) $$\begin{array}{c} \frac{{\tilde{v}}_{j+1}\left(x\right)-{\tilde{v}}_{j}\left(x\right)}{\Delta t}-\left(r-L\right){\tilde{v}}_{j}\left(x\right)=r,\;\;x>h_{j}, \end{array}$$

(81) $$\begin{array}{c} {\tilde{v}}_{j}\left(x\right)=0,\;\;x\leq h_{j}. \end{array}$$

Set *q* = Δ*t* ^−1^ + *r* and $G_{j}={\left(q\Delta t\right)}^{-1}{\tilde{v}}_{j+1}-q^{-1}(r-L)g={\left(q\Delta t\right)}^{-1}{\tilde{v}}_{j+1}-q^{-1}Kr$; then (80) can be rewritten as follows:

(82) $$\begin{array}{c} q^{-1}\left(q-L\right){\tilde{v}}_{j}\left(x\right)=G_{j}\left(x\right),\;\;x>h_{j}. \end{array}$$

Notice that the sequence of problems (81) and (82) has the form (53), where *G*_*j*_(*x*) is monotonically increasing function. Applying a finite difference scheme to (81) and (82) which approximates *q* ^−1^(*q* − *L*) by formulas (14) and (15), we obtain the discrete problem of form (54) which can be easily solved by using (64). See details in Section 3.2.

## 5. Numerical Examples

### 5.1. The FDS&WH Method and the Method of Cont and Voltchkova (2005)

In this subsection we apply our finite difference scheme with Wiener-Hopf method (we refer to the method FDS&WH) to KoBoL process and compare barrier option prices with the results obtained by the method in Cont and Voltchkova (2005) [9] (we refer to this method as* CV method*). We study convergence of two methods for processes of order *ν* < 1 and *ν* > 1.

*Example 1* (Process of order *ν* < 1). To compare CV method with FDS&WH for processes of order *ν* < 1, we take KoBoL model with parameters *σ* = 0, *ν* = 0.5, *λ*_+_ = 4.0, *λ*_−_ = −6.0, and *c* = 1.0. We choose instantaneous interest rate *r* = 0.04879, time to expiry *T* = 0.5 year, strike price *K* = 100, and the barrier *H* = 90. As the base finite difference scheme we choose the one developed in [12].

In Table 1, we compare the down-and-out barrier put option prices calculated by FDS&WH and CV methods for spot prices *S* = 91,101,111,121 (the values are obtained on a PC with characteristics AMD Turion (tm) 64X2 1.6 GHz, 896 Mb, under Windows XP). We see that FDS&WH method demonstrates very fast convergence: in few seconds the accuracy reaches less than 0.5%. In the same time CV method converges very slowly and gives an error in 2-3% only after several hours of calculations. From Table 1 we clearly see that prices computed by FDS&WH stabilize sufficiently fast, while the ones computed by CV method essentially vary from the previous space step. Notice that near the barrier the prices computed by CV method are especially unstable.

*Example 2* (Process of order *ν* > 1). In the case of processes of order *ν* > 1, we take KoBoL model with parameters *σ* = 0, *ν* = 1.2, *λ*_+_ = 8.8, *λ*_−_ = −14.5, and *c* = 1. We choose riskless rate *r* = 0.04879, time to expiry *T* = 0.1 year, strike price *K* = 100, and the barrier *H* = 80.

In Table 2, we compare the down-and-out barrier put option prices calculated by FDS&WH and CV methods. The FDS method can be extended for the case of infinite variation (*ν* > 1); see details in [32]. CV method demonstrates better convergence in comparison with the previous example, but FDS&WH method converges faster, especially in the neighborhood of the barrier.

### 5.2. The FDS&WH Method and FDS (Levendorskiǐ et al. (2006)), [12]

In this subsection we apply our finite difference scheme with Wiener-Hopf method to KoBoL process and compare American option prices with the results obtained by the finite difference method in [12] (we refer to this method as *FDS method*). We take KoBoL model with parameters *σ* = 0, *ν* = 0.2, *λ*_+_ = 3.2, *λ*_−_ = −5.4, and *c* = 1. We choose riskless rate *r* = 0.03, time to expiry *T* = 0.5 year, and strike price *K* = 100. The differences between prices and early exercise boundaries computed by the both methods are insignificant.  Table 3 confirms our observation. As we see form Table 3 the time of computation by FDS&WH method is in several times lesser.

## 6. Conclusion

Many option pricing problems can be solved by using finite difference schemes. The method is very popular in practice, because, in a diffusion model, the correspondent system has a tridiagonal matrix which can be easily inverted.

In the presence of jumps, we have the additional integral term which can be replaced by a discrete sum. As the result, one needs to invert a dense Toeplitz matrix. To avoid this problem, many authors (see, e.g., [9–11]) suggest computing the integral part by using the solution from the previous time step, while the differential term is treated implicitly. This leads to the explicit-implicit scheme, with tridiagonal system which can be solved in *O*(*M*ln⁡*M*) operations, where *M* is a number of space discretization points. However, the advantage in speed turns to the drawback in accuracy, especially in the case of barrier options. In the infinite activity case described in [9], the explicit-implicit scheme demonstrates bad convergence near the barrier and hence also becomes time consuming (see details in [5]).

In [12], an accurate implicit finite difference scheme for pricing American options was developed. The procedure of inversion for the dense matrix of the system is iterative, and it requires 5–10 iterations on each time step. Hence, for a fixed space and time steps, modification of the scheme for barrier options is in several times slower than that of the scheme in [9], but more accurate as examples in [5] show.

In [33], the case of discrete monitoring is considered. The usual backward recursion that arises in discrete barrier option pricing is converted into a set of independent integral equations by using a *z*-transform approach. In order to solve these equations, the rectangle quadrature rule transforms each integral equation into a Toeplitz linear system which is solved by iterative algorithms as in [12].

In the paper, we suggest a new approach which incorporates the Wiener-Hopf factorization method into a finite difference scheme with a Toeplitz system. Notice that our algorithm has the same complexity as the ones which use the explicit-implicit scheme, with a tridiagonal matrix. However, our method is more accurate, because it inverts the whole Toeplitz matrix, not only its tridiagonal part.

We give an important probabilistic interpretation based on the infinitely divisible distributions theory to the Laurent operators in the Wiener-Hopf factorization identity for finite difference schemes. It implies very useful properties which allow developing effective methods for solving many standard problems on option pricing (e.g., European, barrier, first touch digital, and American options).

## Figures and Tables

**Table:** (a)

Parameters		CV			FDS & WH		
Δ*x*	*N*_*τ*_	Option price	*ϵ*	CPU time, sec.	Option price	*ϵ*	CPU time, sec.
0.001	93	0.0577	−59.1%	1	0.1380	−2.2%	1
0.0005	152	0.0716	−49.2%	3	0.1400	−0.8%	2
0.00025	253	0.0873	−38.1%	19	0.1408	−0.2%	8
0.0001	520	0.1073	−24.0%	78	0.1411	0.0%	33
0.00005	926	0.1197	−15.2%	324	0.1411	0.0%	126
0.000025	1688	0.1281	−9.2%	1348	0.1411		568
0.00001	4000	0.1330	−5.7%	14655			

**Table:** (b)

Parameters		CV			FDS & WH		
Δ*x*	*N*_*τ*_	Option price	*ϵ*	CPU time, sec.	Option price	*ϵ*	CPU time, sec.
0.001	93	0.2344	−19.8%	1	0.2899	−0.8%	1
0.0005	152	0.2464	−15.7%	3	0.2915	−0.2%	2
0.00025	253	0.2571	−12.0%	19	0.2920	0.0%	8
0.0001	520	0.2679	−8.3%	78	0.2922	0.0%	33
0.00005	926	0.2740	−6.2%	324	0.2922	0.0%	126
0.000025	1688	0.2787	−4.6%	1348	0.2922		568
0.00001	4000	0.2832	−3.1%	14655			

**Table:** (c)

Parameters		CV			FDS & WH		
Δ*x*	*N*_*τ*_	Option price	*ϵ*	CPU time, sec.	Option price	*ϵ*	CPU time, sec.
0.001	93	0.2182	−16.7%	1	0.2583	−1.4%	1
0.0005	152	0.2273	−13.3%	3	0.2604	−0.6%	2
0.00025	253	0.2353	−10.2%	19	0.2614	−0.3%	8
0.0001	520	0.2434	−7.1%	78	0.2618	−0.1%	33
0.00005	926	0.2481	−5.3%	324	0.2620	0.0%	126
0.000025	1688	0.2517	−4.0%	1348	0.2621		568
0.00001	4000	0.2552	−2.6%	14655			

**Table:** (d)

Parameters		CV			FDS & WH		
Δ*x*	*N*_*τ*_	Option price	*ϵ*	CPU time, sec.	Option price	*ϵ*	CPU time, sec.
0.001	93	0.1718	−15.4%	1	0.1995	−1.7%	1
0.0005	152	0.1781	−12.2%	3	0.2012	−0.9%	2
0.00025	253	0.1838	−9.4%	19	0.2022	−0.4%	8
0.0001	520	0.1896	−6.6%	78	0.2027	−0.2%	33
0.00005	926	0.1930	−4.9%	324	0.2029	−0.1%	126
0.000025	1688	0.1956	−3.6%	1348	0.2030		568
0.00001	4000	0.1981	−2.4%	14655			

KoBoL parameters: *σ* = 0, *ν* = 0.5, *λ*_+_ = 4, *λ*_−_ = −6, *c* = 1.

*K* = 100, *H* = 90, *r* = 0.04879, *T* = 0.5, *ϵ*: the relative difference between the current option price and the price computed by FDS & WH method for space step Δ*x* = 0.000025.

(a): *S* = 91; (b): *S* = 101; (c): *S* = 111; (d): *S* = 121.

**Table:** (a)

Parameters		CV			FDS & WH		
Δ*x*	*N*_*τ*_	Option price	*ϵ*	CPU time, sec.	Option price	*ϵ*	CPU time, sec.
0.001	95	0.372	−28.9%	1	0.551	5.3%	1
0.0005	218	0.402	−23.3%	2	0.543	3.6%	3
0.00025	501	0.431	−17.7%	10	0.536	2.3%	15
0.0001	1501	0.464	−11.4%	188	0.529	1.0%	98
0.00005	3440	0.481	−8.2%	1045	0.526	0.4%	482
0.000025	7882	0.492	−6.1%	5520	0.524		2697

**Table:** (b)

Parameters		CV			FDS & WH		
Δ*x*	*N*_*τ*_	Option price	*ϵ*	CPU time, sec.	Option price	*ϵ*	CPU time, sec.
0.001	95	2.314	−5.7%	1	2.565	4.5%	1
0.0005	218	2.344	−4.5%	2	2.522	2.7%	3
0.00025	501	2.369	−3.5%	10	2.494	1.6%	15
0.0001	1501	2.394	−2.5%	188	2.471	0.7%	98
0.00005	3440	2.407	−1.9%	1045	2.461	0.3%	482
0.000025	7882	2.418	−1.5%	5520	2.455		2697

**Table:** (c)

Parameters		CV			FDS & WH		
Δ*x*	*N*_*τ*_	Option price	*ϵ*	CPU time, sec.	Option price	*ϵ*	CPU time, sec.
0.001	95	2.304	−3.4%	1	2.428	1.8%	1
0.0005	218	2.320	−2.7%	2	2.414	1.2%	3
0.00025	501	2.335	−2.1%	10	2.403	0.7%	15
0.0001	1501	2.349	−1.5%	188	2.393	0.3%	98
0.00005	3440	2.357	−1.2%	1045	2.389	0.1%	482
0.000025	7882	2.364	−0.9%	5520	2.386		2697

**Table:** (d)

Parameters		CV			FDS & WH		
Δ*x*	*N*_*τ*_	Option price	*ϵ*	CPU time, sec.	Option price	*ϵ*	CPU time, sec.
0.001	95	1.547	−2.2%	1	1.567	−0.9%	1
0.0005	218	1.554	−1.8%	2	1.575	−0.4%	3
0.00025	501	1.560	−1.4%	10	1.579	−0.2%	15
0.0001	1501	1.566	−1.0%	188	1.581	0.0%	98
0.00005	3440	1.570	−0.7%	1045	1.581	0.0%	482
0.000025	7882	1.573	−0.5%	5520	1.581		2697

**Table:** (e)

Parameters		CV			FDS & WH		
Δ*x*	*N*_*τ*_	Option price	*ϵ*	CPU time, sec.	Option price	*ϵ*	CPU time, sec.
0.001	95	0.862	−1.5%	1	0.849	−3.0%	1
0.0005	218	0.865	−1.2%	2	0.861	−1.7%	3
0.00025	501	0.867	−0.9%	10	0.868	−0.9%	15
0.0001	1501	0.870	−0.6%	188	0.872	−0.3%	98
0.00005	3440	0.872	−0.4%	1045	0.874	−0.1%	482
0.000025	7882	0.873	−0.3%	5520	0.875		2697

KoBoL parameters: *σ* = 0, *ν* = 1.2, *λ*_+_ = 8.8, *λ*_−_ = −14.5, *c* = 1.

*K* = 100, *H* = 80, *r* = 0.04879, *T* = 0.1; *N*_*τ*_: number of time steps; *ϵ*: the relative difference between the current option price and the price computed by FDS & WH method for space step Δ*x* = 0.000025.

(a): *S* = 81; (b): *S* = 91; (c): *S* = 101; (d): *S* = 111; (e): *S* = 121.

**Table 3:** American put, time of computation: FDS & WH versus FDS.

Parameters		Relative difference		Time of computation, sec.	
Space step Δ*x*	Number of time steps *N*_*τ*_	*ϵ*_*p*_	*ϵ*_*b*_	FDS	FDS & WH
0.002	65	0.21%	0.2%	14	1
0.001	112	0.21%	0.2%	64	3
0.0005	203	0.2%	0.2%	536	13

KoBoL parameters: *σ* = 0, *ν* = 0.2, *λ*_+_ = 3.2, *λ*_−_ = −5.4, *c* = 1.

*K* = 100, *r* = 0.03, *T* = 0.5.

*ϵ*_*p*_ and *ϵ*_*b*_ are the maximums of the relative differences between correspondent prices and boundaries, respectively, in the region *S* ≤ 1.3*K*.
